# Not only mutations but also tumorigenesis can be substantially attributed to DNA damage from reactive oxygen species in *RUNX1::RUNX1T1*-fusion-positive acute myeloid leukemia

**DOI:** 10.1038/s41375-022-01752-5

**Published:** 2022-11-11

**Authors:** Jeffrey D. Mandell, J. Nick Fisk, Ethan Cyrenne, Mina L. Xu, Vincent L. Cannataro, Jeffrey P. Townsend

**Affiliations:** 1 Program in Computational Biology and Bioinformatics, Yale University, New Haven, CT USA; 2 Department of Biostatistics, Yale School of Public Health, New Haven, CT USA; 3 Department of Pathology, Yale School of Medicine, New Haven, CT USA; 4 Department of Biology, Emmanuel College, Boston, MA USA; 5 Department of Ecology and Evolutionary Biology, Yale University, New Haven, CT USA

**Keywords:** Cancer genomics, Oncogenesis

## To the Editor

Gunnarsson et al. [1] examined fusions in pediatric acute myeloid leukemia (AML), ascertained their association with COSMIC single-base substitution signatures (SBS), and identified one enrichment: SBS18 in *RUNX1::RUNX1T1*-fusion-positive cases. Having demonstrated that SBS18 is a substantial source of mutations in *RUNX1::RUNX1T1*-fusion-positive AML cases, the authors suggested that DNA damage caused by reactive oxygen species (ROS)—the proposed etiology of SBS18—“may be of particular importance in *RUNX1::RUNX1T1*-fusion-positive AML” [1]. Indeed, the *RUNX1::RUNX1T1* fusion disrupts hematopoietic differentiation [2], a process which also takes regulatory input from ROS [3]. However, the proportion of mutational burden attributable to a given mutational process does not necessarily indicate the degree to which the process drives oncogenesis [4, 5]. To assess the contribution of SBS18-associated ROS to these AML cases, one must first quantify the extent to which SBS18-attributable mutations drive oncogenesis; this quantification can be performed with metrics such as cancer effect size [4].

**Fig. 1:**
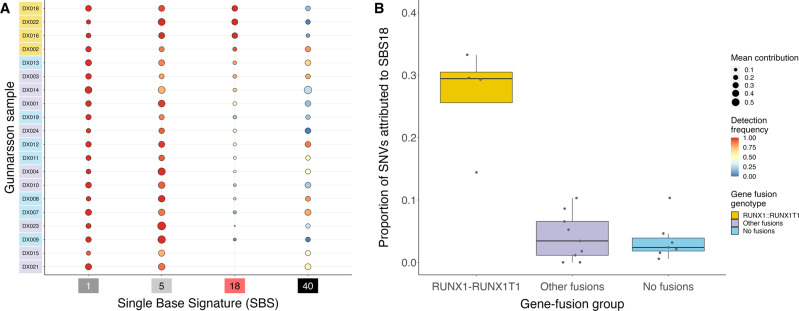
Bootstrapped mutational signature analysis and SBS18 attributions by fusion genotype. **A** Mean fraction of a sample’s SNVs that are attributed to a signature (circle size) across 100 bootstraps for each Gunnarsson et al. [1] sample and signature and the fraction of bootstraps in which the signature was detected (warmer colors: higher fractions). The depicted signatures—SBS1, SBS5, SBS18, and SBS40—account for 87.5% of the total attribution of SNVs to signatures; no other signature accounts for more than 3% of the attribution. **B** Proportion (jittered points), medians (crossbar), 25% and 75% quartiles (column bottoms and tops) of sample SNVs attributed to SBS18 in cancers with RUNX1::RUNX1T1 fusions (yellow), other fusions (mauve), and no fusions (light blue) in the Gunnarsson et al. [1] whole-genome sequence samples.

To attribute SNVs reported by Gunnarsson et al. [1] to the subset of COSMIC v3.2 mutational signatures that have been deemed relevant to AML [6] and that are not associated with prior treatment (as all samples collected were pediatric and diagnostic), we executed MutationalPatterns [7] signature extraction 100 times on resampled trinucleotide mutation profiles and reported mean values. We found SBS1 (an aging signature), SBS5 (a clock-like signature also associated with age), and SBS18 (usually attributed to ROS) to be the most substantial contributors to somatic mutational burden, largely in agreement with Gunnarsson et al. [1] (Gunnarsson et al. [1] didn’t identify a contribution from SBS5, perhaps due to signature bleeding [8]). *RUNX1::RUNX1T1*-fusion-positive samples exhibited an elevated SBS18 signature that was stable across bootstraps (Fig 1A): a significantly higher proportion of mutations in these samples was attributable to SBS18 than in samples with other fusions or no fusions (*P* < 0.01 for both comparisons, Mann–Whitney U test; Fig. 1B).

To assess the contributions of SBS18 and other signatures not only to mutational burden but also to oncogenesis in each Gunnarsson sample, we first estimated the cancer effect sizes of AML somatic variants using a combined dataset of the 20 whole-genome Gunnarsson samples plus an additional 197 whole-exome samples from TCGA [9]. The cancer effect size is a measure that quantifies the evolutionary advantage—namely, increased survival or proliferation—that is conferred by single-nucleotide variants. The observation of a somatic variant in tumor sequence data depends jointly on the baseline rate at which mutations arise in viable cancer cells and on the magnitude of benefit those mutations confer to cancer cell proliferation and survival. The baseline mutation rates were determined per-sample using the regression framework from dNdScv [10] that estimates gene mutation rates at the cohort level. Specific rates for each variant were rescaled based on the sample-specific trinucleotide mutation profile.

We used the cancereffectsizeR package to perform mutation rate estimation and to calculate the maximum-likelihood cancer effect sizes of all single-nucleotide variants under its default model of selection [4]. We then attributed proportions of the effect size of each variant to mutational signatures based on how likely each corresponding mutagenic process was to have caused that variant in a viable cancer cell (i.e., in accordance with the trinucleotide contexts of the variants, the trinucleotide profiles defining each signature, and the relative presence of each signature in samples carrying the variants). Computing these attributions for each signature across all variants, we obtained estimates of the proportion of total cancer effect attributable to each signature (cancer effect weight) [11].

**Fig. 2:**
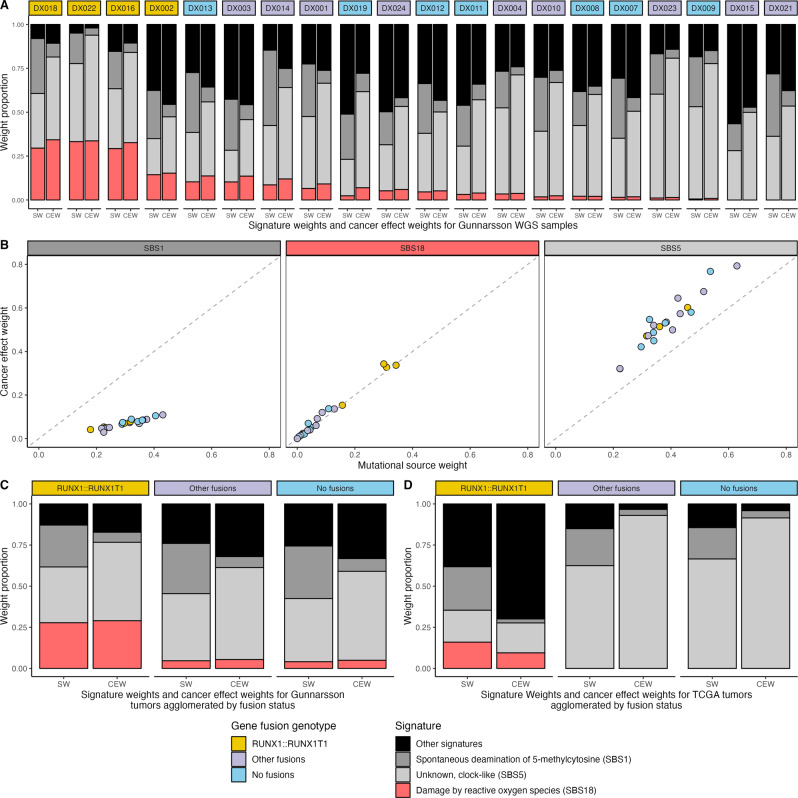
Bar plots and scatter plots of signature weight and cancer effect proportions in acute myeloid leukemias with *RUNX1::RUNX1T1* fusions, with other fusions, and with no fusions, in whole-genome sequenced samples from Gunnarsson et al. [1] and a TCGA whole-exome dataset with its SNVs agglomerated across cases. **A** Proportional stacked bar plots of signature weights (SW) and cancer effect weights (CEW) for 20 cases whose cancers were whole-genome sequenced by Gunnarsson et al. [1] labeled by fusion genotype (*RUNX1::RUNX1T1*-fusion-positive, yellow box; other fusions, mauve box; and no fusions, light blue box). **B** Scatter plots of cancer effect weight versus mutational source weight in the Gunnarsson samples for SBS1 (age-associated cytidine deamination), SBS18 (DNA damage caused by reactive oxygen species), and SBS5 (an unknown age-associated signature). **C** Proportional stacked bar plots of signature weights (SW) and cancer effect weights (CEW) averaged across the Gunnarsson et al. [1] samples. **D** Proportional stacked bar plots of signature weights (SW) and cancer effect weights (CEW) for the agglomerated set of SNVs from 197 TCGA cases whose cancers were whole-exome sequenced.

On a case-by-case basis across the Gunnarsson cohort, mutations attributed to SBS18 consistently contributed to cancer effect as well as mutational burden (Fig. 2A). Other signatures varied in this respect: mutations attributed to aging-related cytidine deamination (SBS1) contributed substantially less to cancer effect than they did to mutational burden, and mutations attributed to another age-related process (SBS5) contributed substantially more (*P* < 0.001, two-sample Wilcoxon signed-rank test; Fig. 2B). To an extent that verged on significance, SBS18 contributed more to oncogenesis than to mutational burden (*P* = 0.06, two-sample Wilcoxon signed-rank test). Per tumor, substantially higher proportions of both mutational signature weights and cancer effect weights were attributed to SBS18 in *RUNX1::RUNX1T1*-fusion-positive cases than other cases (Fig. 2A–B).

To further validate the increased oncogenic effect of SBS18 mutations in *RUNX1::RUNX1T1*-fusion-positive cases in the Gunnarsson cohort, we separately analyzed the cancer effect weights of mutational sources in the TCGA cohort [9]. Because AMLs have low mutational burdens and whole-exome sequencing reveals few mutations per case, we performed signature analysis on a cohort-wide set of mutations derived from all of the *RUNX1::RUNX1T1*-fusion-positive cases, all of the cases with other fusions, and all of the cases with no fusions. Concordant with a parallel analysis of the Gunnarsson samples (Fig. 2C), the TCGA *RUNX1::RUNX1T1* fusion samples demonstrated higher signature weights and cancer effect weights as a consequence of SBS18 than did cases with other fusions or cases with no fusions (Fig. 2D). This result validates our demonstration that in *RUNX1::RUNX1T1*-fusion-positive AML cases, ROS-associated processes contribute not only mutations but also oncogenic effects, supporting further investigation into the prevention of these processes.

## Data Availability

Somatic variant calls from whole-genome-sequenced pediatric AML cases were acquired from the Gunnarsson group and can be requested from the group via https://figshare.com/s/5a1ca3f39611c39bfaae. Additional somatic variant data were acquired from cBioPortal and are publicly available under the identifier aml_tcga_pan_can_atlas_2018. All other data and code used in the analyses are available at https://github.com/Townsend-Lab-Yale/sbs18_aml/.

## References

[1] Gunnarsson R, Yang M, Olsson-Arvidsson L, Biloglav A, Behrendtz M, Castor A (2021). Single base substitution mutational signatures in pediatric acute myeloid leukemia based on whole genome sequencing. Leukemia..

[2] Al-Harbi S, Aljurf M, Mohty M, Almohareb F, Ahmed SOA (2020). An update on the molecular pathogenesis and potential therapeutic targeting of AML with t(8;21)(q22;q22.1);RUNX1-RUNX1T1. Blood Adv.

[3] Sillar JR, Germon ZP, DeIuliis GN, Dun MD. The role of reactive oxygen species in acute myeloid leukaemia. Int J Mol Sci. 2019;20, 10.3390/ijms20236003. 10.3390/ijms20236003 PMC6929020 31795243

[4] Cannataro VL, Gaffney SG, Townsend JP (2018). Effect Sizes of Somatic Mutations in Cancer. J Natl Cancer Inst.

[5] Cannataro VL, Gaffney SG, Sasaki T, Issaeva N, Grewal NKS, Grandis JR (2019). APOBEC-induced mutations and their cancer effect size in head and neck squamous cell carcinoma. Oncogene..

[6] Alexandrov LB, Kim J, Haradhvala NJ, Huang MN, Tian Ng AW, Wu Y (2020). The repertoire of mutational signatures in human cancer. Nature..

[7] Blokzijl F, Janssen R, van Boxtel R, Cuppen E (2018). MutationalPatterns: comprehensive genome-wide analysis of mutational processes. Genome Med.

[8] Maura F, Degasperi A, Nadeu F, Leongamornlert D, Davies H, Moore L (2019). A practical guide for mutational signature analysis in hematological malignancies. Nat Commun.

[9] The Cancer Genome Atlas Research Network. Genomic and epigenomic landscapes of adult de novo acute myeloid leukemia. N Engl J Med. 2013;368:2059–74. 10.1056/NEJMoa1301689 PMC3767041 23634996

[10] Martincorena I, Raine KM, Gerstung M, Dawson KJ, Haase K, Van Loo P (2017). Universal patterns of selection in cancer and somatic tissues. Cell..

[11] Cannataro VL, Mandell JD, Townsend JP. Attribution of cancer origins to endogenous, exogenous, and preventable mutational processes. Mol Biol Evol. 2022;39, 10.1093/molbev/msac084. 10.1093/molbev/msac084 PMC9113445 35580068

